# Living HTA: Automating Health Technology Assessment with R

**DOI:** 10.12688/wellcomeopenres.17933.1

**Published:** 2022-07-21

**Authors:** Robert A. Smith, Paul P. Schneider, Wael Mohammed

**Affiliations:** 1 School of Health and Related Research, University of Sheffield, Sheffield, S1 4DA, UK; 2 Lumanity, Sheffield, S1 2GQ, UK; 3 Dark Peak Analytics, Sheffield, S11 7BA, UK

**Keywords:** HEOR, HTA, APIs, R, plumber

## Abstract

**Background:** Requiring access to sensitive data can be a significant obstacle for the development of health models in the Health Economics & Outcomes Research (HEOR) setting. We demonstrate how health economic evaluation can be conducted with minimal transfer of data between parties, while automating reporting as new information becomes available.

**Methods:** We developed an automated analysis and reporting pipeline for health economic modelling and made the source code openly available on a GitHub repository. The pipeline consists of three parts: An economic model is constructed by the consultant using pseudo data. On the data-owner side, an application programming interface (API) is hosted on a server. This API hosts all sensitive data, so that data does not have to be provided to the consultant. An automated workflow is created, which calls the API, retrieves results, and generates a report.

**Results:** The application of modern data science tools and practices allows analyses of data without the need for direct access – negating the need to send sensitive data. In addition, the entire workflow can be largely automated: the analysis can be scheduled to run at defined time points (e.g. monthly), or when triggered by an event (e.g. an update to the underlying data or model code); results can be generated automatically and then be exported into a report. Documents no longer need to be revised manually.

**Conclusions:** This example demonstrates that it is possible, within a HEOR setting, to separate the health economic model from the data, and automate the main steps of the analysis pipeline.

## Introduction

The development of economic models generally involves the transfer of sensitive data (e.g. individual patient or price data) between parties. This paper demonstrates how the use of application programming interfaces (API) allows data-owners in the Health Economics & Outcomes Research (HEOR) industry to collaborate with multiple partners on health economic decision models, while, retaining full control of their data. The use of an API furthermore makes it possible to streamline and automate reporting as new information becomes available, significantly reducing the financial and administrative burden of economic model updates.

To our knowledge this is the first publication to outline a process for automated reporting in HEOR, which we term Living HTA, and the first to demonstrate the process of sending health economic model algorithms to sensitive data using APIs.

Two other bodies of work are particularly relevant. The first is the OpenSafely initiative, which inspired this work. Williamson *et al.* ^1^ describe the OpenSafely interface, which was developed to analyse electronic health records data without the need to share confidential patient information:

“secure software interface that allows detailed pseudonymized primary care patient records to be analysed in near-real time where they already reside - hosted within the highly secure data centre of the electronic health records vendor — to minimize the reidentification risks when data are transported off-site”.

The method described in this paper has a similar objective, but aims to protect sensitive information in the HEOR sector.

The second work, a publication by Adibi *et al.* ^2^, describes a cloud-based model accessibility platform for models developed in R. The authors make the case for cloud based platforms to improve the accessibility, transparency and standardization of health economic models, particularly highlighting the benefits of hosting computationally burdensome models on remote servers. The authors outline a framework for hosting models, contained within R packages, which are run using calls to an API. A set of standardized model call functions provide the user of the API with enough information to pass the necessary parameters to the model, run the model, and retrieve the necessary results directly into an R session. The publication is the first, to our knowledge, to discuss the enormous implications that remote model hosting could have in the HEOR industry.

We combine elements from both Adibi *et al.* ^2^ and the OpenSafely initiative, and provide an open-source code base which demonstrates the ease with which APIs can be deployed on remote servers to avoid the need to share sensitive data, and enabling automation of model updates. In short, we propose that data owners (e.g. pharmaceutical companies or governments), with support from health economists, host their own model accessibility platforms.

Our hope is that providing these materials will encourage others to use these methods to improve the transparency, accessibility and efficiency of health economic models.

## Methods

We developed an automated analysis and reporting pipeline for health economic modeling. It consists of three parts:

- An economic model. The model can initially be developed using pseudo data – that is, randomly generated data, which has the same format as the actual data, but does not contain any sensitive information.
- An API, hosted on the company or data provider side. It can be generated using the R package plumber. An automated workflow is created. This workflow sends the economic model to the company API. The model is then run within the company server. The results are sent back to the consultant, and a (PDF) report is automatically generated using RMarkdown ^3^. This API server hosts all sensitive data, so that data does not have to be sent between parties.
- All of these processes can be controlled with a web-based user-interface. We provide an example user-interface built in the R shiny package ^4^, based on the tutorial application in our previous paper ^5^. This application allows users to select input parameters with which to query the API, and view the results. This allows non-technical stakeholders to interact with the model in real time, while allowing the company to retain control of the data. The application will always reflect the data on the company server, and the model hosted by the consultant at the time of use.

**Figure 1.**
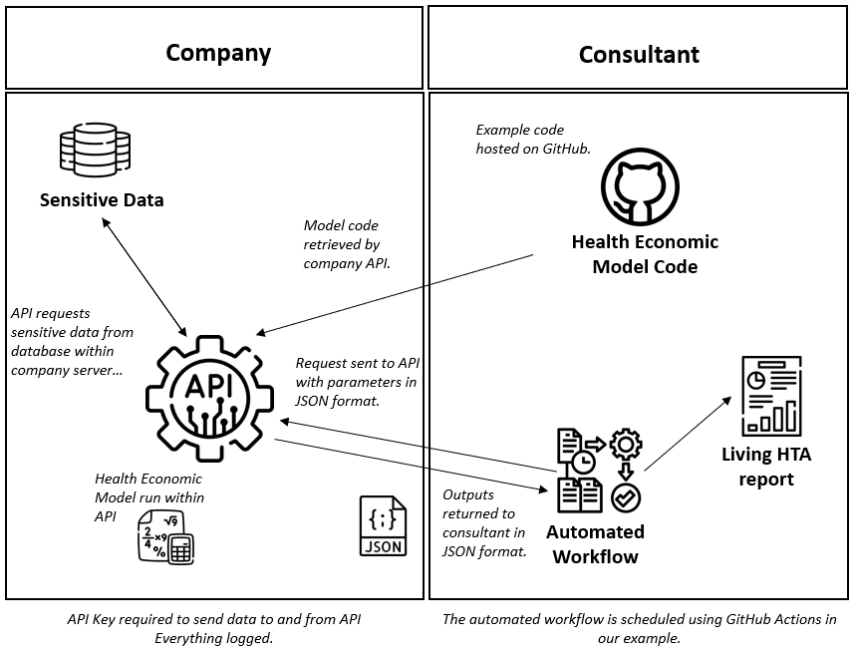
Schematic showing the interaction between the company API (application programming interface) and the consultant automated workflow. HTA, Health Technology Assessment; JSON, Javascript Object Notation.

Figure 1 shows a schematic of the interaction between the company API and the consultant automated workflow. All of the methods discussed in this paper, as well as the code for the demonstration app can be found contained within an open access GitHub repository (see *Software availability* ^6^).

### The economic model

This model code has been adapted from the Decision Analysis in R for Technologies in Health (DARTH) group’s open source Cohort state-transition model (the Sick-Sicker Model) which is discussed in Alarid-Escudero *et al.* ^7^ with open source code available online ^8^. The code includes several functions, but for the purpose of this example we can treat the model as a black box, as a single function called *run_model* which runs the DARTH Sick Sicker model. The *run_model* function takes a single argument, *psa_inputs*, which is a data-frame containing Probabilistic Sensitivity Analysis parameter inputs for the model variables that are allowed to vary.

The data-frame has four columns:

- *parameter* - the name of the parameter (e.g. p_HS1)
- *distribution* - the distribution of that parameter (e.g. “beta”)
- *V1* - the first parameter for the distribution in R (for beta this would be *shape1*, for normal this would be *mean*)
- *V2* - the second parameter for the distribution in R (for beta this would be *shape2*, for normal this would be *sd*)

The *run_model* function returns a data-frame with six columns. The first three columns are costs for each treatment option, and the second three columns are Quality Adjusted Life Years (QALYs) for each treatment option. Each row represents the result of the model run for a set of inputs.

### The API

An application programming interface is a set of rules, in the form of code, that allow different computers to interact with one another in real time. Whereas user-interfaces such as those generated by the R package *shiny* allow humans to interact with data, APIs are designed to enable computers to interact with data ^4^.

When a ‘client’ application wants to access data, it initiates an API call ( *request*) via a web-server, to retrieve the data. If this request is deemed valid, the API makes a call to an external program/server, the server sends a response to the API with the data, and the API transfers the data to the ‘client’ application. In a sense, the API is the broker (or middle-man) between two systems.

There are numerous benefits to APIs:

- in supporting programmatic access. In contrast to what web applications offer (for example shiny apps), APIs allow users to access data, or other utilities (for example, proprietary applications) programmatically. Programmatic access enables users to invoke actions through an application or third-party tool. For example, R users can write a function that fetches or analyses data via an API and use it in their workflow as any other user-defined function.
- in allowing cross-platform communications. Statisticians and decision-model developers can use different programming languages or packages. For example, APIs can allow a decision analytic model, developed in C++ to programmatically utilise data from a bayesian meta-analysis performed using the Python programming language.
- in aiding speed of collaboration between institutions, ensuring inputs and outputs are standardised so that applications can ‘talk’ to one another. Users from one institution need not to take into account the software or package used by their partners, but focus on how they would interact with the expected data.
- in security, eliminating the necessity to share data manually (e.g. via email). All interaction with data can be logged and access can be restricted by passwords and by limiting IP address access. For example, APIs can safely allow statisticians to programmatically accumulate sub-group summary-statistics from securely stored trial-data to inform a network meta-analysis.
- in expanding sharing avenues. For example, APIs can allow institutions to give limited access to their proprietary tools such as in-house decision-analytic models. Users of such tools can pass their data to the model and receive the respective outputs via the API.
- eliminating computational burden on the client side (since all computation is done on the API owner side).

There are lots of different implementations of APIs, but the main focus of this paper is on *Partner APIs*, which are created to allow data transfer between two different institutions. This requires a medium level of security, usually through the creation of access keys that are shared with partners.

In the examples below we use Javascript Object Notation (JSON), a data interchange format that is commonly used to transfer information between computers, to pass information to and from our API. Since the model is written in R, we convert back and forth between JSON and R data formats using the *jsonlite* R package ^9^.

### Creating the API using plumber

The R package *plumber* allows programmers to create web APIs by decorating R source code with roxygen-like comments ^10, 11^. These functions are then made available as API endpoints by plumber.

The API can be called using a number of HTTP request methods (also known as HTTP verbs). The most-commonly used methods POST, GET, PUT, PATCH, and DELETE correspond to create (POST and PUT), read (GET), update (PATCH), and delete (DELETE) operations. These annotations generate the API’s endpoint(s) and specify the operation(s) or response(s) the respective R function is responsible for generating. The below example shows the ‘GET’ request (the default for web-browsers).

The code below gives an example function which echos a message. The function takes one input, a string with the message, and outputs the message contained within a list. If this function was created in R it would return a list containing some text, like this: The message is: ‘example_msg’.


1	#* Echo back the input
2	#* @param msg The message to echo
3	#* @get /echo 
4	function(msg="") {
5	  list(msg = paste0("The message is: '", msg, "'"))
6	}


 The code for the model function uses the same principles, but is much more developed. There are three arguments to the model API; *path_to_psa_inputs*, *model_functions* and *param_updates*.

The core API function created by plumber sources the model functions from software development website GitHub, obtains the model parameter data from within the API, and then overwrites the rows of the parameter updates that exist in *param_updates*. It then runs the model functions using the updated parameters, post-processes the results, checks that no sensitive data is included in the results, and then returns a data-frame of results. This entire process occurs in the server on which the API is hosted, with inputs and outputs passed to the API over the web in JSON format.

***Code chunk 1 - Generating the API***


 1	library(dampack)
 2	library(readr)
 3	library(assertthat)
 4	
 5	#* @apiTitle Client API hosting sensitive data
 6	#*
 7	#* @apiDescription This API contains sensitive data, the client does not
 8	#* want to share this data but does want a consultant to build a health
 9	#* economic model using it, and wants that consultant to be able to run
10 	#* the model for various inputs
11 	#* (while holding certain inputs fixed and leaving them unknown).
12 	
13 	#* Run the DARTH model
14 	#* @serializer csv
15 	#* @param path_to_psa_inputs is the path of the csv file containing the PSA parameters
16 	#* @param model_functions gives the GitHub repository to source the model code
17 	#* @param param_updates gives the replacement values of the editable parameters
18 	#* @post /runDARTHmodel
19 	function(path_to_psa_inputs = "parameter_distributions.csv",
20 	          model_functions = paste0("https://raw.githubusercontent.com/",
21 	                                      "BresMed/plumberHE/main/R/darth_funcs.R"), 
22 	          param_updates = data.frame(
23 	            parameter = c("p_HS1", "p_S1H"),
24 	            distribution = c("beta", "beta"),
25 	            v1 = c(25, 50),
26 	            v2 = c(150, 70)
27	          )) {
28 
29 
30 	  # source the model functions from the shared GitHub repo... 
31 	  source(model_functions)
32 	
33 	  # read in the csv containing parameter inputs
34 	  psa_inputs <- as.data.frame(readr::read_csv(path_to_psa_inputs))
35 	
36 	  # for each row of the data-frame containing the variables to be changed... 
37 	  for(n in 1:nrow(param_updates)){
38 	
39 	  # update parameters from API input
40 	  psa_inputs <- overwrite_parameter_value(
41 	                               existing_df = psa_inputs,
42 	                               parameter = param_updates[n,"parameter"], 
43 	                               distribution = param_updates[n,"distribution"], 
44 	                               v1 = param_updates[n,"v1"],
45 	                               v2 = param_updates[n,"v2"])
46 	  }
47 	
48 	  # run the model using the single run-model function.
49 	  results <- run_model(psa_inputs)
50 	
51 	  # check that the model results being returned are the correct dimensions
52 	  # here we expect a single dataframe with 6 columns and 1000 rows
53 	  assertthat::assert_that(
54 	    all(dim(x = results) == c(1000, 6)), 
55 	    class(results) == "data.frame",
56 	    msg = "Dimensions or type of data are incorrect,
57 	  please check the model code is correct or contact an administrator.
58 	  This has been logged"
59 	  )
60 	
61 	  # check that no data matching the sensitive csv data is included in the output
62 	  # searches through the results data-frame for any of the parameter names,
63 	  # if any exist they will flag a TRUE, therefore we assert that all = F
64 	  assertthat::assert_that(all(psa_inputs[, 1] %in%
65 	        as.character(unlist(x = results,
66 	                               recursive = T)) == F))
67 	
68 	  return(results)
69 	
70 	}


### Deploying an API

There are numerous providers of cloud computing services. The most convenient, yet not the cheapest, service is offered by RStudio Connect. An account is required for this, but once you have one it is possible to deploy the API directly from the Rstudio IDE. RStudio have a blog on how to publish an API created using plumber to RStudio connect here.

### Interacting with the API

We first show how to run the model from an R script, calling the API and retrieving the results of the model run. We then show how to use GitHub actions to automate the process, running the R script when triggered by an event (e.g. a data-update) or a scheduled time (e.g. the 1st of each month).

***Interact with the API from an RScript.*** We use the *POST* function from the *httr* package to query the API ^12^ - as shown in the code chunk below. This function requires an internet connection. We provide values for several arguments:

- *url* - the URL of the RStudio Connect server hosting the API we have created using plumber.
- *path* - the path to the API within the server URL.
- *query* & *body* - objects passed to the API in list format, with names matching the plumber function arguments.
- *config* - allows the user to specify the KEY needed to access the API.

The *content* function attempts to determine the correct format for the output from the API based upon the content type. This function ensures that the result object is a dataframe.

The script then then goes on to save the data and generate a PDF report from the outputs using the RMarkdown package ^3^, the code for which can be found here. The R-Markdown report uses functions adapted from the *darkpeak* R package.

***Code chunk 2 - Query the API, retrieve model results and generate report***


 1	# remove all existing data from the environment.
 2	rm(list = ls())
 3	
 4	library(ggplot2)
 5	library(jsonlite)
 6	library(httr)
 7	
 8	# run the model using the connect server API
 9	results <- httr::content( 
10 	  httr::POST(
11	    # the Server URL can also be kept confidential, but will leave here for now
12	    url = "https://connect.bresmed.com",
13	    # path for the API within the server URL
14	    path = "rhta2022/runDARTHmodel",
15	    # code is passed to the client API from GitHub.
16	    query = list(model_functions =
17                           paste0("https://raw.githubusercontent.com/",
18	                             "BresMed/plumberHE/main/R/darth_funcs.R")),
19	    # set of parameters to be changed ...
20	    # we are allowed to change these but not some others
21	    body = list(
22	      param_updates = jsonlite::toJSON( 
23	        data.frame(parameter = c("p_HS1","p_S1H"),
24                           distribution = c("beta","beta"), 
25                           v1 = c(25, 50),
26                           v2 = c(150, 100))
27	      )
28	    ),
29	    # we include a key here to access the API here the key is a env variable
30	    config = httr::add_headers(Authorization = paste0("Key ",
31	                                                             Sys.getenv("CONNECT_KEY")))
32	  )
33	)
34
35	# write the results as a csv to the outputs folder...
36	write.csv(x = results,
37	           file = "outputs/darth_model_results.csv")
38	 
39	source("report/makeCEAC.R")
40	source("report/makeCEPlane.R")
41	 
42	# render the markdown document from the report folder,
43	# passing the results dataframe to the report.
44	rmarkdown::render(input = "report/darthreport.Rmd",
45	                     params = list("df_results" = results),
46	                     output_dir = "outputs")


***Living HTA - scheduling model report updates.*** Once the API is created and hosted online, it can be called any time. The advantage of this is that any updates to either the model code, or the data used by the model, can be undertaken separately and the model re-run by either party. Calls to the API can also be scheduled at routine intervals. This would enable the health economic evaluation model report to be updated, without human interaction, at regular intervals to reflect the most up-to-date data.

In the example below we show how a GitHub Actions (other providers available) workflow can be used to automate an update to a health economic evaluation ^13^. The workflow runs at 0:01 on the first day of every month or any time there are changes made to the source code. It first clones the GitHub repository on a GitHub actions Windows 2019 server, then install the necessary dependencies, before running the script described above to generate the model report. It creates a pull request to the repo with this new updated report. If GitHub is not the preferred location of report storage, it is possible to send the report via email or save to cloud storage solutions such as Google Drive or Dropbox.

***Code chunk 3 - Automated report updates***


 1	on:
 2	  push:
 3	    branches:
 4	    - main
 5	  schedule:
 6	    - cron: '1 1 1 * *'
 7	
 8	name: Run DARTH model on client API 
 9	jobs: 
10 	  createPullRequest: 
11 	    runs-on: windows-2019 
12	    env: 
13	      GITHUB_PAT: ${{ secrets.GITHUB_TOKEN }}
14	 # Load repo and install R 
15	   steps: 
16	   - uses: actions/checkout@master
17	   - uses: r-lib/actions/setup-r@master
18	 
19	   - name: Setup pandoc
20	     uses: r-lib/actions/setup-pandoc@v2
21	     with:
22	       pandoc-version: '2.17.1.1'
23	 
24	   - name: Install TinyTeX
25	     uses: r-lib/actions/setup-tinytex@v2 
26	     env:
27	          # install full prebuilt version 
28	          TINYTEX_INSTALLER: TinyTeX
29	 
30	   - name: Install dependencies 
31	     run: |
32	          install.packages(
33	          c("reshape2", "jsonlite", "httr", "readr", "rmarkdown", "markdown")
34	          )
35	          install.packages(
36	          "scales", dependencies = TRUE, repos = 'http://cran.rstudio.com/'
37	          )
38	          install.packages(
39	          "ggplot2", dependencies = TRUE, repos = 'http://cran.rstudio.com/'
40	          )
41	     shell: Rscript {0}
42	 
43	   - name: Run the model from API and create report 
44	     env:
45	         CONNECT_KEY: ${{secrets.PLUMBER_SECRET}}
46	     run: |
47	         source("scripts/run_darthAPI.R")
48	     shell: Rscript {0}
49	 
50	   - name: Create Pull Request
51	     uses: peter-evans/create-pull-request@v3
52	     with:
53	       token: ${{ secrets.GITHUB_TOKEN }}
54	       commit-message: Automated Model Run from API
55	       title: 'Living HTA Automated Model Run' 
56	       body: >
57	         Automated model run
58	         labels: report, automated pr



## Results

All source code for the API, the economic model, the automated model update framework, and the example dataset are available online (see *Software availability* ^6^ and *Underlying data* ^14^).

The most up to date automated report, based on the data held on the exemplar API (hosted on RStudio Connect), can always be found here.

The method has been validated by two co-authors using Windows and MAC with example data (see *Underlying data* ^14^). Those validating the method were able to run the model with updated parameter values without access to sensitive data, were able to trigger the automated report generation based on existing sensitive data, and were able to query the model through an example R-Shiny application, hosted on GitHub (see *Software availability* ^6^). However we are keen to validate the method further, and invite collaboration. A live exemplar API is currently hosted by Lumanity (using the exact source code provided open access). If the reader is interested to test the functionality of the API please contact the corresponding author, who can provide the key.

## Discussion

As the collection and storage of large data sets has become more commonplace in health & health care settings, this data is increasingly being used to inform decision making. However, concerns about the security of this data, and the ethical implications about linked data sets, make the owners of this valuable resource particularly reluctant to share data with health economic modelling teams. The ability to host APIs on data-owners’ servers, and send the model to the data rather than the data to the model, is one potential solution to this problem. The example described in this paper may be relatively simple, but gives a tech savvy health economist everything they need to set up a modelling framework which does not rely on the sharing of data by a company (or other data-owner).

The framework described has a number of benefits.

- Firstly, no data needs to leave the data-owner’s server. This is likely to significantly reduce administrative burden for both the company and the consultant, and reduce the number of data-leaks.
- Separating the data from the model has significantly improved the transparency of the health economic model. Allowing others to critique methods & hidden structural assumptions, test the code and identify bugs should improve the quality of models in the long run. It also enables the pool of people working on developing the health economic model and accompanying user-interface to be widened, without concern for confidentiality & data security. For example a shiny application could be developed for a model built under this framework without the programmer needing access to any sensitive data or information.
- The computational burden of the model is handled on a remote server. The power of these servers is typically considerably greater than that of a typical personal computer, speeding up model run time considerably. This is likely to be especially important for models that incorporate uncertainty through monte-carlo sampling algorithms which can be parallelized on machines with multiple cores ^15^, for example probabilistic one way sensitivity analysis ^16^ or partial expected value of perfect information ^17^.
- The use of APIs to perform distinct tasks can improve interoperability within the field of health economics. Different modules, or tasks within a modelling framework can be written in different languages (e.g. R, Python, Julia & C++) and linked using APIs. This is likely to improve collaboration between different sub-disciplines, which often use different languages (e.g. health economists in R and data-scientists in Python).
- API calls can be made at any time, and will always reflect the data held by the data owner. In many cases these datasets are updated regularly, allowing companies, and other stakeholders, to see the results of the decision model based on the most up to date data, without needing human intervention to: send new datasets, re-run analysis, write a report, and provide that report in a suitable format for the company. Automating model updates at set schedules, or when data is updated, may be invaluable where data is updated regularly, as has been the case throughout the COVID-19 pandemic.
- Any model can be passed to the API, as long as the inputs and outputs to the model meet the requirements of the API. This means that multiple health economic models could be passed to the API, to be run using the data on the company server, and compared to account for structural uncertainty.

However, the framework has a number of limitations:

- Firstly, the method is relatively complex, and requires a strong understanding of health economic modelling in R, API creation and hosting, RMarkdown or other automated reporting packages, and GitHub Actions. While we hope that this paper provides a useful resource to health economists seeking to utilise these methods, the bulk of the industry still operates in MS Excel ^18^. Providing tuition to upskill health economists, or creating teams consisting of both health economists and data-scientists and software engineers may mediate this limitation somewhat. Groups like the R for HTA consortium has the potential to play a crucial role in upskilling the industry.
- There are still likely to be concerns about data security, even with the authentication procedures built in to the API functionality. Collaboration with experts in this field may mediate this significantly, since there is no fundamental reason why health data is any more sensitive, or vulnerable, than the plethora of other data (including banking data) that relies on APIs every day. It will be important to reassure companies that the use of APIs is likely to reduce, not increase the risk of data breaches, and that every interaction with the data can be logged.
- There is a risk that running the model remotely will result in the perception that the model is a ‘black box’. The use of user-interfaces (such as those increasingly being created in *shiny*) to interrogate the model, as well as the increased transparency associated with being able to share code on sites such as GitHub, should reassure stakeholders that this framework is more transparent than the existing spreadsheet based solutions ^19^.
- Often, when building a model, it is helpful to have the underlying data to be able to investigate the data, often through the generation of descriptive statistics. The process of sharing pseudo-data enables modellers to ensure that the models they create conform to the structure of the data input. However, the modeller still needs to be able to write code that is versatile enough to cope with data with unknown distributions ranges and number of observations. This is easily solved, again by improved training and the use of standard packages such as *hesim* and *heemod* ^20, 21^.

The recent working paper by Adibi *et al.* ^2^ has provided a similar call to action, extolling the virtues of the API for decision modelling, and showing how APIs can be used to shift much of the computational burden away from those querying models, making models more accessible. However, there are several limitations to this brilliant paper. Firstly, while the authors outline a framework for making models more transparent and accessible, and describe how they have done this for a number of models using the PRISM server, they do not provide instruction on how to replicate this process. Additionally, while the authors state that “A practical model accessibility platform should be able to protect confidential information such as patient data and confidential pricing” (p6), the framework as described would require companies to give the owners of the model accessibility platform access to their confidential data, or else host the model accessibility platform themselves.

This paper has attempted to address some of these limitations, providing open source code for the creation and deployment of an API with an accompanying automated health economic evaluation update framework. It also provides open source code on two new pieces of additional functionality not previously described elsewhere; firstly it demonstrates how companies can host APIs themselves to negate the need to share data with subject experts, and secondly it demonstrates how model updates can be automated with scheduled workflows run on remote servers.

## Conclusions

This example framework, with accompanying open source code base, demonstrates that it is possible, within a HEOR setting, to separate a health economic model from the data, and automate the main steps of the analysis pipeline. We believe this is the first application of this procedure in the HEOR context, and is certainly the first example to be made open source for the benefit of the wider community. We hope that this framework will improve the transparency of health economic models, reduce the cost and administrative burden of updating models, and increase the speed at which updates can occur.

## Data availability

### Underlying data

Zenodo: Parameter distributions. https://doi.org/10.5281/zenodo.6727629 ^14^.

This project contains the following underlying data:

- parameter_distributions.csv (example dataset for modification. This dataset also sits within the server, with some of the rows marked as non-editable; these are characterised as ’sensitive’ throughout the manuscript. This dataset is edited in ’Code Chunk 2’ to test the API).

Data are available under the terms of the Creative Commons Attribution 4.0 International license (CC-BY 4.0).

## Software availability

Source code available from: https://github.com/ RobertASmithBresMed/plumberHE.

Archived source code at time of publication: https:// doi.org/10.5281/zenodo.6556888 ^6^

License: MIT
