# Supplementary material for: Living HTA: Automating Health Economic Evaluation with R
Source: Wellcome Open Res. 2022 Oct 11;7:194. Originally published 2022 Jul 21. [Version 2] doi: 10.12688/wellcomeopenres.17933.2 (PMC9593025; doi:10.12688/wellcomeopenres.17933.2)
Supplement: Peer review history

# Peer review: Reviewer response for version 2

**DOI:** 10.21956/wellcomeopenres.20423.r52750

**Published:** 2022-10-24

**Authors:** Mohsen Sadatsafavi

**Affiliations:** 1 Faculty of Pharmaceutical Sciences, University of British Columbia, Vancouver, BC, Canada

Thanks for revising the paper so diligently. I enjoyed reading this manuscript and again find the contribution very relevant to the health economics modeling community and related stakeholders. I have very minor/discretionary suggestions:

- The new Figure 1 is very informative (albeit, I suggest using consistent terms [e.g., data-owner under 'Company'] with the text).
- Under 'Deploying API': Perhaps make it clear that if desired the data-owner can host the API on their own company servers (so really keeping the data local) and do not have to use remote cloud-based solutions.
- The paper can benefit from discussing (somewhere in the Discussion section) the security risk the data-owner is exposed to when letting a consultant upload the code on their server. There are indeed solutions to that (e.g., sandboxing strategies specifically provided by Linux servers, virtualization, etc).

Is the rationale for developing the new method (or application) clearly explained?

Yes

Is the description of the method technically sound?

Yes

Are the conclusions about the method and its performance adequately supported by the findings presented in the article?

Yes

If any results are presented, are all the source data underlying the results available to ensure full reproducibility?

Yes

Are sufficient details provided to allow replication of the method development and its use by others?

No

Reviewer Expertise:

Economic Evaluation; Epidemiology; Medical Decision Making

I confirm that I have read this submission and believe that I have an appropriate level of expertise to confirm that it is of an acceptable scientific standard.

# Peer review: Reviewer response for version 1

**DOI:** 10.21956/wellcomeopenres.19871.r51659

**Published:** 2022-09-12

**Authors:** Devin Incerti

**Affiliations:** 1 Head of Data Science, EntityRisk Inc., Princeton, NJ, USA

This is a useful paper that has the potential to enhance the quality of the data used by health economic models. 

 I have several comments related to the discussion section:

1. The authors should clarify how they envision data being used. In this example, parameter estimates are shared by the data-owner. However, this assumes that the data-owner is capable of estimating the parameters. 
2. A more useful workflow might be one in which data-owners share the raw underlying data (e.g., clinical trial data) and the consultant parameterizes the health economic model using that data. The authors rightfully note that "Often, when building a model, it is helpful to have the underlying data to be able to investigate the data, often through the generation of descriptive statistics." However, they understate the significance of this limitation:
   
  - Creating a fully automated workflow to parameterize a model without access to the underlying data is difficult. For instance: it is often helpful to have access to the actual data to (i) identify outliers and possible coding errors, (ii) recode predictors (e.g., by combining categories with small numbers of observations), and (iii) assess the feasibility of a model specification (e.g., ordered logistic regression vs. multinomial logistic regression). 
  - The authors argue that "pseudo data" can help overcome this problem. While mostly true, creating synthetic data is no trivial task and should be noted as a challenge. I would argue that parameterization without access to the actual data will be easier if the pseudo data is more realistic.
3. While the points raised in #2 above are a limitation of the framework, they are also a potential strength. As noted by the first reviewer, the use of pseudo data may reduce perceived bias; e.g., by requiring statistical analyses to be pre-specified and fully automated. Health economic models (and the statistical models that parameterize them) can be set up as "pipelines" (like in machine learning) capable of being run on any dataset and blind to the outcomes.
4. Data-owners may be concerned that the transfer of sensitive data may not be secure. The authors should comment on any potential security concerns.
5. The current code is open-source. The authors may want to comment on closed-source approaches since many consultants will be hesitant to make code publicly available. 

I also have some minor comments about the code and user interface:

- The body of a for loop should be indented (e.g., starting on line 40 in code chunk 1) to improve readability.
- *renv* should be used to manage package dependencies and enhance the reproducibility of the paper. I needed to install *readr* before I could successfully run code chunk 2.
- The user interface does not currently provide error messages if invalid model inputs are used (e.g., if a negative shape parameter is used for utility). This is issue #2 in the GitHub repository, but it has not yet been addressed. 

Is the rationale for developing the new method (or application) clearly explained?

Yes

Is the description of the method technically sound?

Yes

Are the conclusions about the method and its performance adequately supported by the findings presented in the article?

Yes

If any results are presented, are all the source data underlying the results available to ensure full reproducibility?

Yes

Are sufficient details provided to allow replication of the method development and its use by others?

Yes

Reviewer Expertise:

Health economics, Biostatistics

I confirm that I have read this submission and believe that I have an appropriate level of expertise to confirm that it is of an acceptable scientific standard.

# Response

**Published:** 2022-09-26

**Authors:** Robert Smith

**Affiliations:** University of Sheffield, UK

**This is a useful paper that has the potential to enhance the quality of the data used by health economic models.**

 We are glad you think it is useful and thanks for the helpful comments.

**The authors should clarify how they envision data being used. In this example, parameter estimates are shared by the data-owner. However, this assumes that the data-owner is capable of estimating the parameters.**

 We agree, this was kept intentionally vague because different use-cases may require sharing at different stages in the process (potentially companies may wish to do some early analysis ‘in-house’). However, we have added a section in the discussion stating that:

 “In the (intentionally simple) example we give in this paper, it is assumed that the data owner provides readily estimated parameter values. In a real world use case, this does not need to be the case: the consultant may also send a survival model specification, for example, to estimate parameters from individual patient data, before the parameters are then passed to the health economic model.” 

****

**A more useful workflow might be one in which data-owners share the raw underlying data (e.g., clinical trial data) and the consultant parameterizes the health economic model using that data. The authors rightfully note that "Often, when building a model, it is helpful to have the underlying data to be able to investigate the data, often through the generation of descriptive statistics." However, they understate the significance of this limitation:**

1. **Creating a fully automated workflow to parameterize a model without access to the underlying data is difficult. For instance: it is often helpful to have access to the actual data to (i) identify outliers and possible coding errors, (ii) recode predictors (e.g., by combining categories with small numbers of observations), and (iii) assess the feasibility of a model specification (e.g., ordered logistic regression vs. multinomial logistic regression).**
2. **The authors argue that "pseudo data" can help overcome this problem. While mostly true, creating synthetic data is no trivial task and should be noted as a challenge. I would argue that parameterization without access to the actual data will be easier if the pseudo data is more realistic.;**

We agree that building the model without access to the underlying data is very difficult and may not be feasible in many cases - especially where the model is more complex (for example deviating from a typical oncology model). However, lots of use cases remain, including model updates for new data cuts and, increasingly, updates to RWE from databases. It’s still really early days, but we could gradually approach ‘livingHTA’ using hospital data to inform commissioning in a continuous feedback process. 

****

**While the points raised in #2 above are a limitation of the framework, they are also a potential strength. As noted by the first reviewer, the use of pseudo data may reduce perceived bias; e.g., by requiring statistical analyses to be pre-specified and fully automated. Health economic models (and the statistical models that parameterize them) can be set up as "pipelines" (like in machine learning) capable of being run on any dataset and blind to the outcomes.**

 Completely agree, we have added a section in the text in the discussion which reads:

 “Specifying a model without access to and feedback from the actual data may come with its own challenges. However,  if these hurdles are overcome, the benefit is that it enforces a thorough statistical analysis plan by default, in every use case. This helps to avoid biases introduced by stakeholder incentives.”

**Data-owners may be concerned that the transfer of sensitive data may not be secure. The authors should comment on any potential security concerns.**

 We agree, and expect this to be a significant hurdle. We have a section in the text that states that:

 "There are still likely to be concerns about data security, even with the authentication procedures built in to the API functionality. Collaboration with experts in this field may mediate this significantly, since there is no fundamental reason why health data is any more sensitive, or vulnerable, than the plethora of other data (including banking data) that relies on APIs every day. It will be important to reassure companies that the use of APIs is likely to reduce, not increase the risk of data breaches, and that every interaction with the data can be logged."

**The current code is open-source. The authors may want to comment on closed-source approaches since many consultants will be hesitant to make code publicly available.**

 Yes, I suspect many people will want to keep their code private. This is kind of contrary to our intentions (which is to create a situation where it is easier to share the model) so we have added a section in the text that states that:

 “However, if it is necessary to restrict access to model code, it is possible for the API to be passed ‘private’ source code. As keen proponents of open source modeling that was determined to be beyond the scope of this publication.”

**The body of a for loop should be indented (e.g., starting on line 40 in code chunk 1) to improve readability.**

 Thanks, we have asked the editorial team to address this in the paper.

 This has also been updated in the code: 

https://github.com/RobertASmithBresMed/plumberHE/commit/9f567d5c6b79b3d20865a4860f33e2de4151b8d7 

**renv should be used to manage package dependencies and enhance the reproducibility of the paper. I needed to install readr before I could successfully run code chunk 2.**

 Thanks, we should have done this before - the lock file can now be found here: 

 https://github.com/RobertASmithBresMed/plumberHE/blob/main/renv.lock

****

**The user interface does not currently provide error messages if invalid model inputs are used (e.g., if a negative shape parameter is used for utility). This is issue #2 in the GitHub repository, but it has not yet been addressed.**

 We have created an issue: https://github.com/RobertASmithBresMed/plumberHE/issues/29 and are in the process of reviewing a solution to this problem. Many thanks for this suggestion.

# Peer review: Reviewer response for version 1

**DOI:** 10.21956/wellcomeopenres.19871.r51661

**Published:** 2022-08-08

**Authors:** Mohsen Sadatsafavi

**Affiliations:** 1 Faculty of Pharmaceutical Sciences, University of British Columbia, Vancouver, BC, Canada

Smith and colleagues introduce an API framework for developing and implementing health economics models without necessarily sharing the underlying data with model developers. This is facilitated via an Application Programming Interface. The main utility of this framework is separating data from the model. The setup of the platform is that the client (say a pharma company) is commissioning the creation of a health economics model via a consultancy firm. The company is in possession of sensitive and not-sharable data that are used to develop the model. To make things work, the company creates a Web server that securely hosts the data. It optionally provides synthetic data to the consultant to help build the model. Once the model is built, it is sent to the Web server where it sits next to the sensitive data to complete what is needed to run the economic evaluation. It then sends the output back to the consultant and a report is generated. This process is further automated such that the entire process is activated at set intervals or after a change in the model or the underlying data. This pipeline uses several widely adopted standards or services such as JSON for data transfer, Github for haring the model code and automation, RMarkdown, and so on. I really enjoyed reading this paper and found it an important step in the right direction.

 I have several suggestions for improving the exposition.

**Major comments:**

1. The authors should be clearer with their terminology throughout as well as which code is running where. It took this reader several reads to finally (apparently) understand. Suggest making it clear that a client has sensitive data and tasks a consultant for creating a model. Make it clear that the first code chunk runs on the client’s server (next to data) while the second is run on the consultant’s side (if I understood things correctly).
2. “ *When a ‘client’ application wants to access data, it initiates an API call (request) via a web-server, to retrieve the data. If this request is deemed valid, the API makes a call to an external program/server, the server sends a response to the API with the data, and the API transfers the data to the ‘client’ application*.” I found these sentences on top of Page 5 quite confusing. Is not sharing sensitive data whilst being able to run the model not the main point of this platform?
3. The only input that the run_model accepts seems to be the PA dataset. Are there flexibilities around other sets of parameters? What if someone wants to change the time horizon of the model? Similarly, what the investigators consider as the output of the model is restrictive (only costs and QALYs). A health economic model can have other payoffs (mortality, disease incidence) and to this reviewer, the presence of clinical or other payoffs is more norm than an exception. Can they make comments on to what extent these parts can be made more flexible?
4. Suggest making it clear that by sharing the model and keeping the data private, the requirement now is for the model to be open-source. This in itself is sharing IP, and the authors can make it clear that their innovation is that the model is shared not the data, and perhaps claim that oftentimes sharing data is more restrictive.
5. Please make it clear that plumber is a state-based environment and as such, it connects all the consultants to the same R session. The advantage of this approach is that it is ‘live’ such that subsequent function calls can be made to interact with the model. The drawback is that multiple consultants might overwrite each other’s sessions and also keeping R alive, especially with complex models can be problematic for the server. The authors can make a distinction with Adibi *et al.*’s PRISM which uses OpenCPU.

**Minor comments:**

1. Is adhering to the DARTH naming convention absolutely necessary? While using such standards is generally good, is this platform not more generic in nature (e.g., the company and the consultant agreeing on a function call)?
2. In the case study for which code chunks are provided, what data are considered sensitive for illustrative purposes?
3. Why the dataframe representing the probabilistic input should have distributions with two parameters? This will preclude the use of some distributions like generalized gamma that have three or more parameters.
4. One advantage of this platform can be that by making the modeler team use synthetic data, the potential for stakeholder bias is also minimized.
5. Introduction: “ *The development of economic models generally involves the transfer of sensitive data (e.g. individual patient or price data) between parties*”. I think ‘ *generally*’ is too strong here. Perhaps use ‘at times’? Many health economics modeling efforts are based on the literature or publicly available data. Perhaps the authors can restrict this statement to models developed by the industry for their new health technologies for which there are often sensitive data.
6. I could not understand the point of overwrite_parameter_value() function. Can the authors clarify? Similarly, in the code chunk #2, line 52: “ *here we expect a single dataframe with 6 columns and 1000 rows*”. It is not obvious to me how we expect this to be 1000 exactly. Should this not be decided by the dimension of the input data frame?
7. Page 7: “ *for this, but once you have one it is possible*”; ‘ *you*’ is a bit colloquial for a science paper. Similarly, referring to Adibi’s framework as ‘ *brilliant*’ is a bit colloquial.

Is the rationale for developing the new method (or application) clearly explained?

Yes

Is the description of the method technically sound?

Yes

Are the conclusions about the method and its performance adequately supported by the findings presented in the article?

Yes

If any results are presented, are all the source data underlying the results available to ensure full reproducibility?

Yes

Are sufficient details provided to allow replication of the method development and its use by others?

No

Reviewer Expertise:

Economic Evaluation; Epidemiology; Medical Decision Making

I confirm that I have read this submission and believe that I have an appropriate level of expertise to confirm that it is of an acceptable scientific standard, however I have significant reservations, as outlined above.

# Response

**Published:** 2022-09-26

**Authors:** Robert Smith

**Affiliations:** University of Sheffield, UK

**Smith and colleagues introduce an API framework for developing and implementing health economics models without necessarily sharing the underlying data with model developers. This is facilitated via an Application Programming Interface. The main utility of this framework is separating data from the model. The setup of the platform is that the client (say a pharma company) is commissioning the creation of a health economics model via a consultancy firm. The company is in possession of sensitive and not-sharable data that are used to develop the model. To make things work, the company creates a Web server that securely hosts the data. It optionally provides synthetic data to the consultant to help build the model. Once the model is built, it is sent to the Web server where it sits next to the sensitive data to complete what is needed to run the economic evaluation. It then sends the output back to the consultant and a report is generated. This process is further automated such that the entire process is activated at set intervals or after a change in the model or the underlying data. This pipeline uses several widely adopted standards or services such as JSON for data transfer, Github for having the model code and automation, RMarkdown, and so on. I really enjoyed reading this paper and found it an important step in the right direction.**

 Thank you for taking the time to review this paper and for the very constructive comments which we think have improved the quality of the paper. We are glad that you enjoyed reading it and think that it is taking an important step in the right direction.

**The authors should be clearer with their terminology throughout as well as which code is running where. It took this reader several reads to finally (apparently) understand. Suggest making it clear that a client has sensitive data and tasks a consultant for creating a model. Make it clear that the first code chunk runs on the client’s server (next to data) while the second is run on the consultant’s side (if I understood things correctly).**

 We agree. We have done several things to try and address this issue - we believe this makes the paper clearer:

1. We have changed references to ‘client’ in the main body of the text to ‘data-owner’ to reduce confusion with the term client in the context of APIs.
2. We have added a section in the methods section of the manuscript which reads as below. This hopefully gives more context. making it clear that the data-owner has the data and is tasking the consultant to build the model without access to the data.
3. Third, we have added additional information to the code chunk headings. For “Code chunk 1 - Generating the API”  we have added, “this code is run on the data-owner’s server”. For “Code chunk 2 - Query the API, retrieve model results and generate report” we have added, “this code is run by the consultant". 

In the methods section:

 “A common problem in health technology assessment is a situation in which a data-owner (e.g. a company in the pharmaceutical industry) holds sensitive data, but requires the services of a consultant to conduct health economic modelling. For example the data-owner may have interim clinical trial data at the patient level, and may need an external health economist (the consultant) to build a state transition model to determine the cost-effectiveness of the treatment. Currently the data-owner may be required to send the consultant cuts of data as the trial progresses (e.g. 12 month, 18 month, 24 month). This is burdensome and results in multiple iterations of sharing sensitive data. We propose a solution that does not require the sharing of data between parties and allows for automated updates to the analysis as data is updated. This automated analysis and reporting pipeline for health economic modelling consists of three parts:”

**“When a ‘client’ application wants to access data, it initiates an API call (request) via a web-server, to retrieve the data. If this request is deemed valid, the API makes a call to an external program/server, the server sends a response to the API with the data, and the API transfers the data to the ‘client’ application.” I found these sentences on top of Page 5 quite confusing. Is not sharing sensitive data whilst being able to run the model not the main point of this platform?**

 Thank you, we agree that the description of the process for an API is too general and not specific to this use case. We have changed this paragraph to read as below, with the hope that this is clearer: 

 “An application programming interface is a set of rules, in the form of code, that allow different computers to interact with one another in real time. Whereas user-interfaces such as those generated by the R package shiny allow humans to interact with data, APIs are designed to enable computers to interact with each other in a consistent way (for example to exchange data or access algorithms) 4.

 When a request is sent by one application (an API call) via a web server to another application, it is evaluated and if appropriate, an action is triggered and/or a response is sent. An API call can contain anything, ranging from data to a model, or just a single GET request (for example a web browser sends a request when trying to access a web page). If necessary, the API transfers the response of the API request to the client application (the application making the request). In a sense, the API is the broker (or middle-man) between two systems.

 In the example described in this paper, the consultant is going to send an API request to the data owner's computer. The request will contain some data (non-sensitive model inputs) and the health economic model, which is to be run on the data owner's computer using the sensitive data available there. The API runs the health economic model and responds to the request with the output of the model.” 

**The only input that the run_model accepts seems to be the PA dataset. Are there flexibilities around other sets of parameters? What if someone wants to change the time horizon of the model? Similarly, what the investigators consider as the output of the model is restrictive (only costs and QALYs). A health economic model can have other payoffs (mortality, disease incidence) and to this reviewer, the presence of clinical or other payoffs is more norm than an exception. Can they make comments on to what extent these parts can be made more flexible?**

 We have added the following paragraph to the paper.

 Also, see response to minor comment #3:

 “The function described is designed as a simple reproducible example. The proposed method is in principle flexible to any inputs, model structure, and outputs.”

****

**Suggest making it clear that by sharing the model and keeping the data private, the requirement now is for the model to be open-source. This in itself is sharing IP, and the authors can make it clear that their innovation is that the model is shared not the data, and perhaps claim that oftentimes sharing data is more restrictive.**

****

 We have adapted the introduction section to read:

 “We therefore see the primary contribution of this paper as being the development of a system in which the health economic model and the data are two separate entities, and the health economic model is sent to the data rather than the other way around. By working in this way, it is theoretically easier to share the model without the sensitive data on which it is run, although making the model open source is not a requirement”.

**Please make it clear that plumber is a state-based environment and as such, it connects all the consultants to the same R session. The advantage of this approach is that it is ‘live’ such that subsequent function calls can be made to interact with the model. The drawback is that multiple consultants might overwrite each other’s sessions and also keeping R alive, especially with complex models can be problematic for the server. The authors can make a distinction with Adibi et al.’s PRISM which uses OpenCPU.**

 We agree with your comment, but think it is unlikely to be a problem given that most platforms create multiple R processes which I believe gives a similar result to PRISM. For example, a plumber-powered API could be containerised and deployed on Google Cloud Run, which scales the resources (number of API containers) based on demand (https://w-mohammed.github.io/posts/deploying-a-plumber-API-on-Google-Cloud/). It is also maybe a very specific technical issue that can be overcome in isolation to the wider method and concept.

 However, the comment is really interesting, and actually, I wonder if the bigger limitation is actually that this means that different R processes are working in isolation, so model runs will not interact, which would be important for things like expert elicitation but can easily be overcome using external databases.

 I hope that the addition of the paragraph below, with reference to PRISM, to the discussion helps to clear some of this up:

 “R is single threaded, and therefore will only work on one task at a time. This can make it slow when lots of requests are made simultaneously, which may occur if a model takes a long time to run. Most hosting platforms (including RSConnect, where we have deployed this example) solve this problem by creating multiple R processes, which work in isolation. The PRISM solution outlined by Abidi et al. [2] uses OpenCPU which works in much the same way. This is fine for the example model we provide, since our model doesn’t store any information required by other users, who would be working on another R process, during the session. Further information can be found on the plumber guide here: https://www.rplumber.io/articles/hosting.html.” 

**Minor**

**Is adhering to the DARTH naming convention absolutely necessary? While using such standards is generally good, is this platform not more generic in nature (e.g., the company and the consultant agreeing on a function call)?**

 The DARTH sick-sicker model has been used as an example because it is a commonly used teaching tool in Health Economics and there are published papers describing the code, so if interested readers want to dig into the code further they can. However, you are completely correct that this process would work for any model. We actually see one of the benefits of this technology being that several consultancies could submit their models to the data - and the results could be compared to better understand the structural uncertainty associated with the models - all without any of the consultancies accessing the underlying data.

**In the case study for which code chunks are provided, what data are considered sensitive for illustrative purposes?**

 The underlying data can be found here: plumberHE/parameter_distributions.csv at main · RobertASmithBresMed/plumberHE (github.com)  

 The sensitive rows of the dataframe are flagged by the column ‘editable’ being FALSE. To give more context around this we have added to the section ‘The economic model’, by adding to the end of the paragraph reading “psa_inputs, which is a data-frame containing Probabilistic Sensitivity Analysis parameter inputs for the model variables that are allowed to vary.” the sentence “Additional, sensitive parameters (including treatment costs and hazard ratio for treatment B) are not allowed to be varied by the API request and will be informed by the values held by the  data-owner.”

**Why the dataframe representing the probabilistic input should have distributions with two parameters? This will preclude the use of some distributions like generalized gamma that have three or more parameters.**

 We agree. However, we sought to make this example as simple as possible. Models constructed using this framework are completely flexible to adapt to any data format or model structure. To reflect this we have added to the bottom of ‘The Economic Model’ section the paragraph:

 “The function described is designed as a simple reproducible example. The proposed method is in principle flexible to any inputs, model structure, and outputs.”

**One advantage of this platform can be that by making the modeler team use synthetic data, the potential for stakeholder bias is also minimized.**

 We agree. The following paragraph was added to the paper:

 “Specifying a model without access to and feedback from the actual data may come with its own challenges. However,  if these hurdles are overcome, the benefit is that it enforces a thorough statistical analysis plan by default, in every use case. This helps to avoid biases introduced by stakeholder incentives.”

**Introduction: “The development of economic models generally involves the transfer of sensitive data (e.g. individual patient or price data) between parties”. I think ‘generally’ is too strong here. Perhaps use ‘at times’? Many health economics modeling efforts are based on the literature or publicly available data. Perhaps the authors can restrict this statement to models developed by the industry for their new health technologies for which there are often sensitive data.**

 We have changed the sentence to read:

 “The development of economic models sometimes involves the transfer of sensitive data (e.g. individual patient or price data) between parties”.

**I could not understand the point of overwrite_parameter_value() function. Can the authors clarify? Similarly, in the code chunk #2, line 52: “here we expect a single dataframe with 6 columns and 1000 rows”. It is not obvious to me how we expect this to be 1000 exactly. Should this not be decided by the dimension of the input data frame?**

 The ‘overwrite_parameter_value’ function takes the data-frame passed by the consultant to the data-owner in code Chunk 2-Line-22, and combines it with the default parameters stored on the company server (it doesn’t overwrite those values, instead creating a new data-frame). This allows the consultant to change any parameter for which they have permission (see point 2 above with the ‘editable’ column).

 In code chunk #1 line 52 the expectation is for a data-frame with 6 columns and 1000 rows. In this simple example, this would have had to be pre-agreed between the consultant and the data-owner since it is hard-coded into the model code, however, the number of PSA runs could be provided as an input to the API if deemed necessary.

**Page 7: “for this, but once you have one it is possible”; ‘you’ is a bit colloquial for a science paper. Similarly, referring to Adibi’s framework as ‘brilliant’ is a bit colloquial.**

 We agree, we have changed page 7 to read:

 “An account is required for this, but provides the benefit of being able to deploy the API directly from the Rstudio integrated development environment.”

 We have also changed the description of the Adibi framework from  “brilliant” to innovative”.

 Thank you again for taking the time to review this paper. We think that these responses and the edits made have improved the clarity of the paper for others.

 Kind regards,

 ​​​​​​​Rob
